# Influence of margin location and luting material on the amount of undetected cement excess on CAD/CAM implant abutments and cement-retained zirconia crowns: an in-vitro study

**DOI:** 10.1186/s12903-019-0809-2

**Published:** 2019-06-14

**Authors:** Peter Gehrke, Konstantin Bleuel, Carsten Fischer, Robert Sader

**Affiliations:** 1 Private Practice for Oral surgery and Implant Dentsitry, Bismarckstraße 27, 67059 Ludwigshafen, Germany; 2 Department of Postgraduate Education, Oral and Dental Medicine, Johann Wolfgang Goethe-University, Frankfurt, Germany; 3 Private Practice, Aschaffenburg, Germany; 4 Sirius Ceramics Laboratory, Frankfurt, Germany; 5 Department for Oral, Cranio-Maxillofacial and Facial Plastic Surgery, Medical Center of the Goethe University Frankfurt, Frankfurt, Germany

**Keywords:** CAD/CAM implant abutments, Cement excess, Cement cleaning, Cement-retained implant restorations, Subgingival margins, Zirconia crowns

## Abstract

**Background:**

The flexibility in designing the submucosal part of CAD/CAM customized implant abutments and the individual positioning of its shoulder line has been suggested to reduce the risk of leaving undetected cement residues, thus preventing adverse effects on peri-implant tissues. A high correlation between excess cement left in the soft tissues and the occurrence of increased biofilm accumulation with sulcular bleeding and/ or suppuration has been reported. This in turn may cause peri-implant inflammation and peri-implant marginal bone loss. The aim of this study was to assess the frequency of cement remnants after the luting of zirconia crowns on CAD/CAM custom molar abutments with different margin levels and to evaluate the impact of the luting material.

**Material and methods:**

A total of 20 titanium molar CAD/CAM implant abutments (BEGO Medical GmbH) with internal taper connection/ internal hex anti-rotation protection, and a convex emergence profile with different margin positions (0, 1, 2 and 3 mm below the mucosa), were virtually designed (Implant Studio, 3Shape) and manufactured. A master cast was scanned, duplicated by a 3D printer and individual gingival masks were produced to simulate peri-implant soft tissues. 20 corresponding zirconia crowns were designed (Cerec 3D, Dentsply Sirona), produced and cemented to the abutments with two different luting materials; a zinc oxide non-eugenol cement (Temp Bond NE) or a methacrylate cement (Panavia V5). To ensure retrievability of the crown/abutment connection, occlusal openings providing access to the abutment screws were designed. Excess cement was thoroughly removed and the crown/abutment units were unscrewed to evaluate the occurrence of cement residues. All the quadrants of each specimen were evaluated for calculation of the ratio between the cement remnant area and the total specimen area using Adobe Photoshop. Spearman analysis was performed to detect correlations between different variables. A two-sided t-test, ANOVA, Mann–Whitney, and Kruskal–Wallis tests were applied to detect differences between the groups.

**Results:**

Cement remnants were found in every depth of the crown abutment complex and in almost every area investigated. The amount of cement residues increased as the crown-abutment margin was located more submucosally. Lingual areas were more prone to cement remnants than other surface areas (*p* = 0.0291). Excess cement was not only found at the margins of the crown-abutment complex, but also underneath (basal) the abutment itself, where cleaning was impossible. No statistical difference in the effect of zinc oxide non-eugenol- and methacrylate cement on the frequency of excess material at the lateral abutment surfaces could be demonstrated in vitro. The proportion of basal abutment aspects covered with cement residues was, however, significantly smaller in Panavia V5 samples with an average of 4.9 ± 3.7% compared to Temp Bond samples with an average of 8.6 ± 5.5%.

**Conclusions:**

Given the results obtained in the present investigation the margin of CAD/CAM molar abutments should be located as coronally as possible to minimize the amount of cement remnants. If an epigingival or supragingival margin location is not feasible due to esthetic concerns, it cannot be recommended to place the margin of molar CAD/CAM abutments deeper than 1.5 mm in the proximal and oral regions.

## Background

An abutment serves as the extension of a dental implant into the oral cavity and thus as basis for the subsequent restoration. Its biological function is to shape and support the peri-implant soft tissues while at the same time functioning as a sufficient barrier for bacterial colonization [1, 2]. Implant abutments are selected according to the bone level, mucosal thickness, angulation, shape and size of the reconstruction. The shoulder margin can be set both sub- and supra-mucosal, depending on soft-tissue architecture and esthetic requirements. Implants can be restored with either screw-retained or cement-retained restorations. The latter is a commonly utilized prosthetic technique since it allows for a tolerance with respect to the implant axis and position and it is familiar to the majority of practitioners [3]. Cemented restorations, however, have a number of disadvantages, including the challenge of completely removing cement remnants around the restoration [4–9]. As undetected cement excess of fixed implant-supported restorations has been associated with clinical and radiographic signs of peri-implant inflammation, there is an essential need to reduce this risk. Agar et al. [10] examined the removal of excess cement around implant crowns with stock abutment margins placed at various levels below an artificial mucosal margin. Even after careful removal attempts, cement remnants could always be detected, independent of the examiners experience or instruments used. Additional aspects, such as the vertical position of the crown-abutment interface or the type of luting material appeared to be influential. These findings are supported by in vitro and in vivo studies demonstrating that the depth of the crown-abutment interface of stock abutments negatively influences the practitioner’s ability to remove cement excess [8, 11, 12]. Another weakness of cement retained restorations is the difficulty or impossibility of removing the restoration in case of complications, without damaging or destroying it. In contrast, the major benefit of screw-retained reconstructions is their retrievability [4, 7]. While the majority of available data refers to prefabricated stock abutments, little is known about the incidence of undetected cement residues of computer-aided designed and manufactured (CAD/CAM) custom abutments in the molar region. The flexibility in designing the submucosal part of custom abutments and the individual positioning of its shoulder line has been suggested to reduce the risk of leaving undetected cement residues, thus preventing adverse effects on peri-implant tissues [13, 14]. However, so far there is little data available to support this hypothesis. Consequently, the aim of the present in vitro study was to assess the frequency of cement remnants after luting of zirconia crowns on convex emergence profile molar CAD/CAM abutments with different margin levels (0, 1, 2 and 3 mm below the mucosa), and to evaluate the impact of two different luting materials.

## Methods

**Fig. 1:**
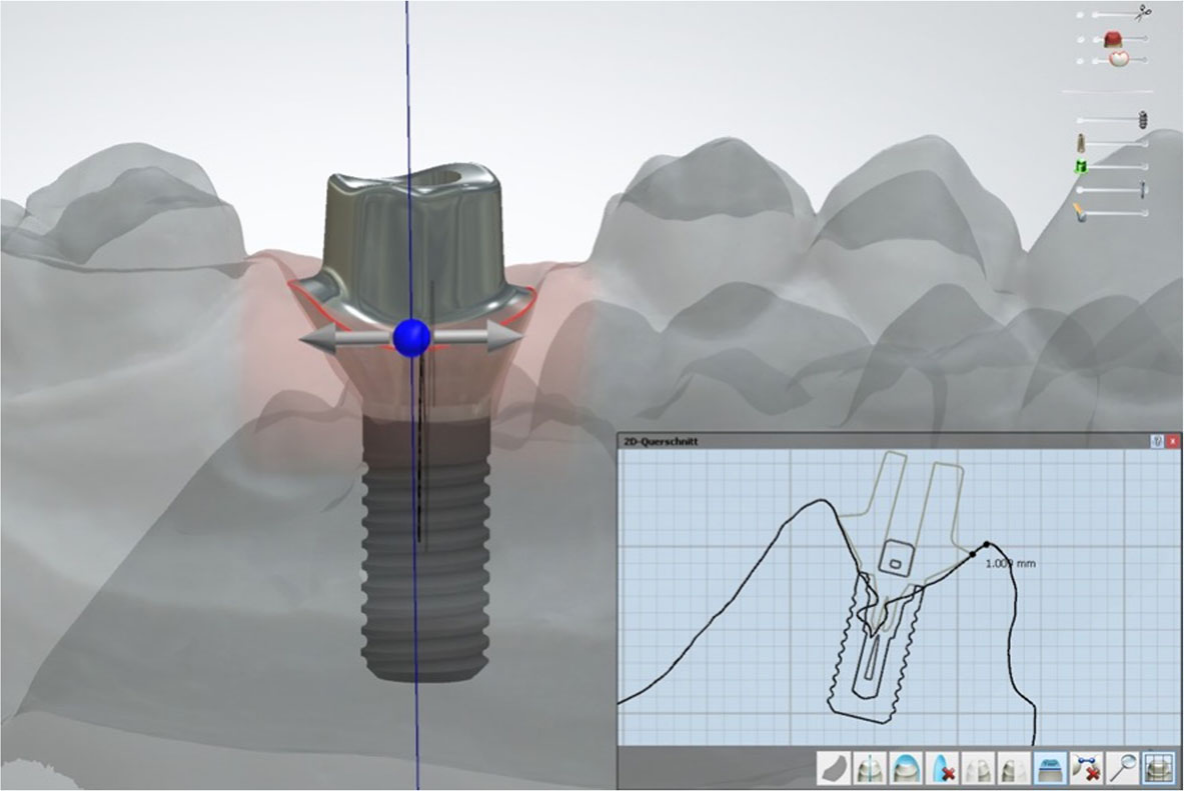
Digital design of abutment emergence profile

**Fig. 2:**
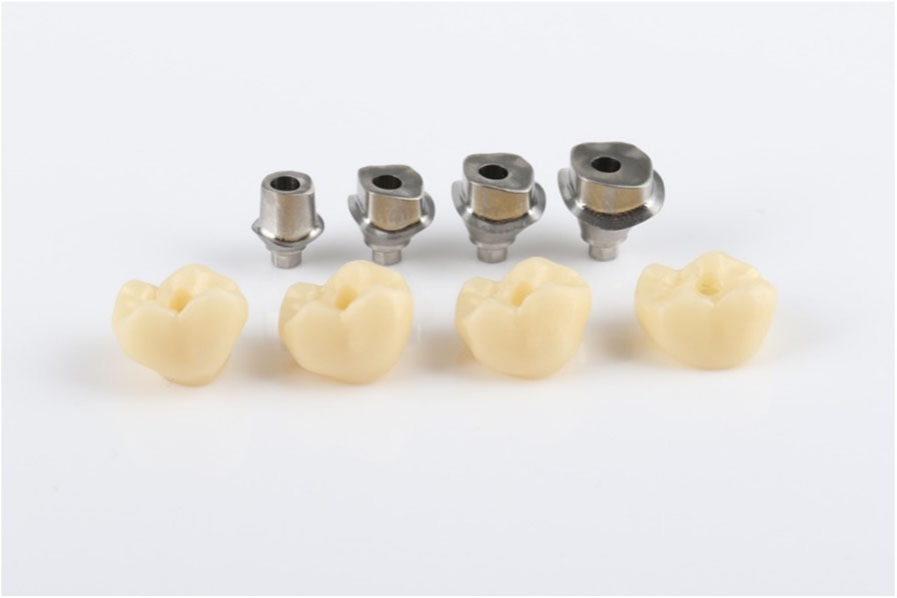
Sample abutments with their corresponding zirconium crowns from left to right: 3 mm–0 mm subgingival

**Fig. 3:**
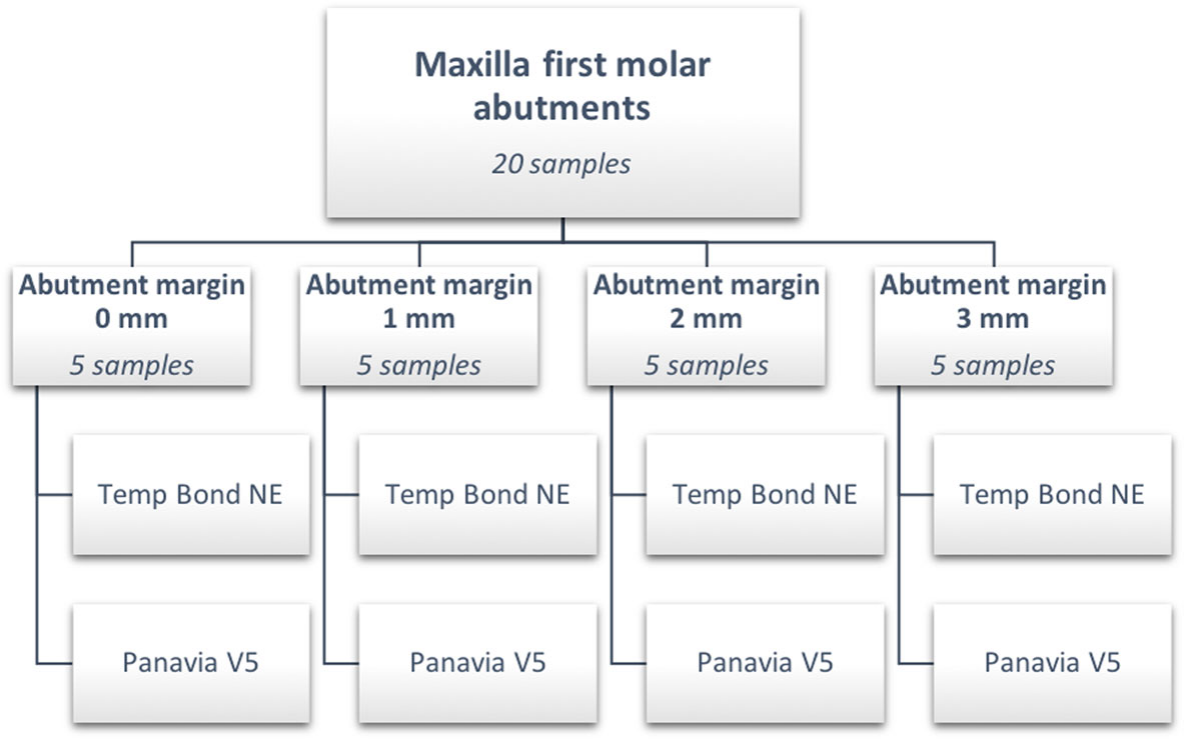
Sample distribution of abutment margin designs and luting materials

**Fig. 4:**
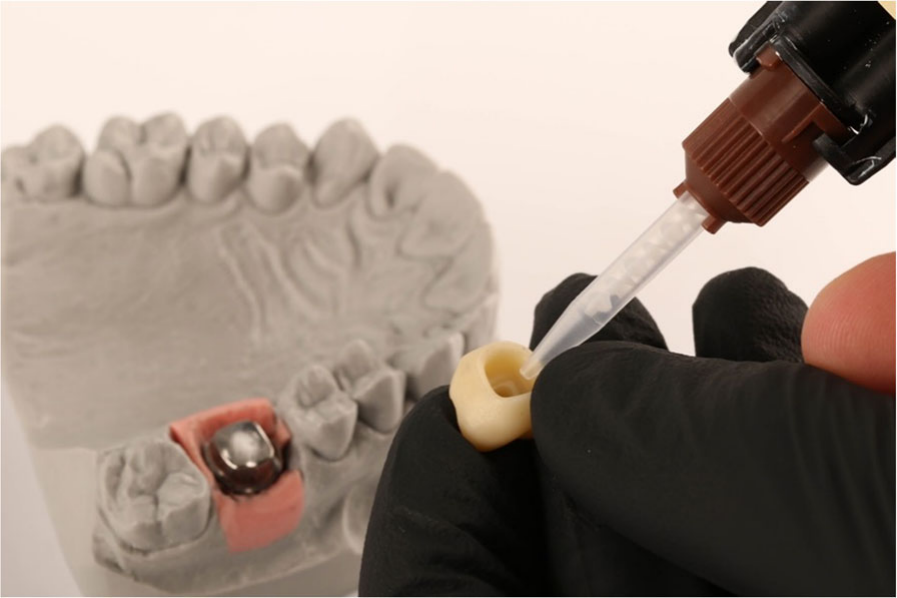
Cementation process on mounted titanium abutment with gingival mask and mixing tip into crown

**Fig. 5:**
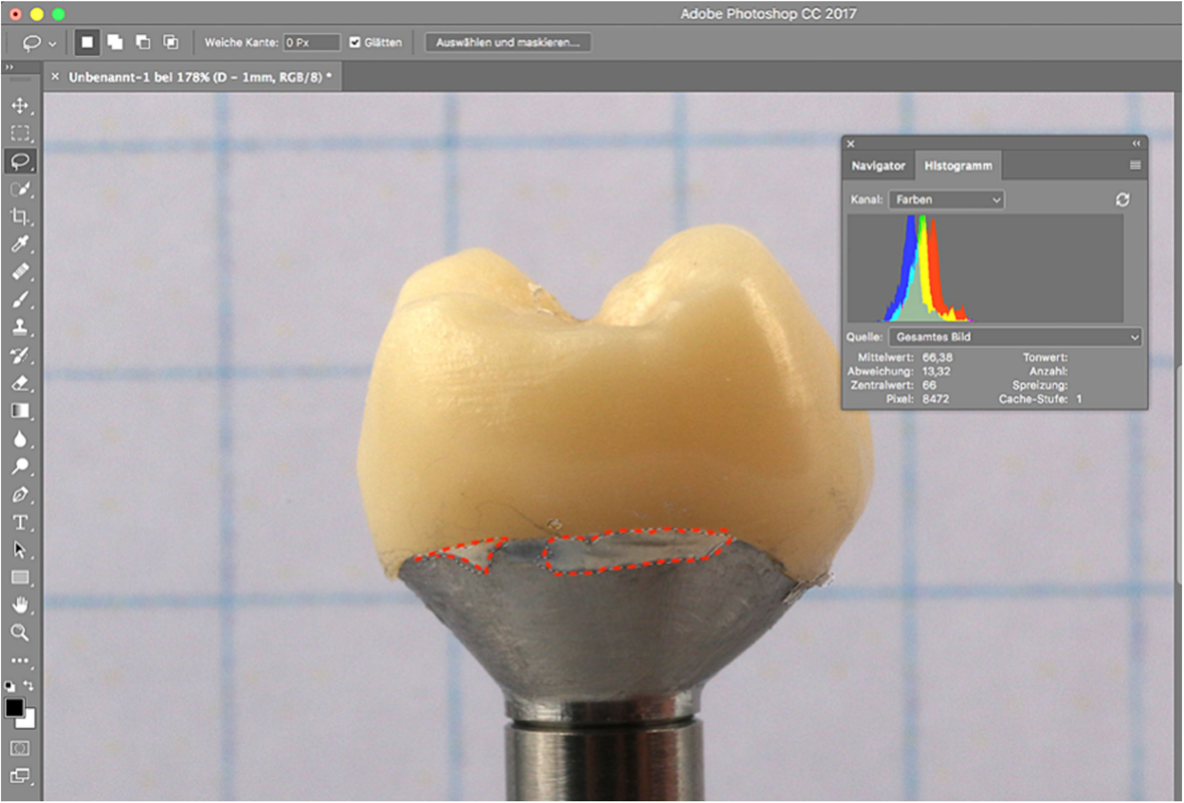
Evaluation of the area covered with cement remnants and the total surface of the specimen in Adobe Photoshop

A total of 20 titanium CAD/CAM implant abutments with a convex emergence profile and different margin positions (0, 1, 2 and 3 mm below the mucosa), were virtually designed (Implant Studio, 3Shape, Copenhagen, Denmark) (Fig. 1) and manufactured (BEGO Medical GmbH, Bremen, Germany). The master cast of a clinical case in which the left maxillary first molar had been replaced by an implant restoration served as the origin of the model. The case was restored using a regular platform two-piece implant with an internal taper connection and internal hex anti-rotation protection (BEGO Semados® RSX D 4.1/ L 11.5, BEGO Implant Systems GmbH & Co. KG, Bremen, Germany). The emergence profile of the peri-implant mucosa had been pre-conditioned by means of a temporary implant-supported single crown. The original master cast with the implant analog (PS IMPA 4.1, BEGO Implant Systems GmbH & Co. KG, Bremen, Germany) was duplicated in type IV plaster (Quadro-Rock Plus, Picodent, Wipperfürth, Germany). All abutment specimens were cleansed three times in an ultrasonic bath at 30 °C for 5 min each, as previously described by the authors [15, 16]. In addition, 20 corresponding monolithic zirconia crowns (Zirlux, Henry Schein, Langen, Germany) were CAD/CAM designed and produced (Cerec 3D, Dentsply Sirona, Bensheim, Germany) (Fig. 2). The master cast with the implant analog was scanned with a 3D scanner (3Shape, Copenhagen, Denmark), duplicated by a 3D printer (Formlabs, Boston, USA) and individual gingival masks were confectioned to simulate the peri-implant mucosal tissues. The light body polyvinyl siloxane gingival mask (Gingifast, Zermak, Marl, Germany) was replicated four times and altered for each depth of the crown-abutment distance. Occlusal openings were designed in the zirconia crowns in order to have access to the occlusal abutment screw after cementation. Before cementation, the top of each prosthetic abutment was covered with a cotton pellet in order to protect the abutment screw. The occlusal crown openings were closed with a dual cured flexible composite Telio CS Link (Ivoclar Vivadent, Liechtenstein) to obturate the screw access and to allow for retrieval of the abutment-crown complex after cementation. The cement was mixed according to the manufacturer’s instructions. A thin uniform layer was applied to all internal surfaces of the crowns by using the mixing tips of the cartridges and seated onto the abutment with constant finger pressure. The exact amount of cement was not quantified as the cementation protocol tried to imitate a real clinical scenario. The cementation process was performed by one experienced clinician (PG). In the first trial, a zinc oxide non-eugenol cement (Temp Bond NE, Kerr Dental, Germany) was used for luting the crowns. After the cleaning and evaluation process, the crown was separated from the abutment and all parts were thoroughly cleaned. For this purpose, the luting area of the CAD/CAM abutments and the inner surfaces of the zirconia crowns were first cleaned from the remaining zinc oxide non-eugenol cement remnants with an acrylic scaler (Hu Friedy Mfg. Co., LLC, Frankfurt, Germany). The inner surfaces of the zirconia crowns were additionally treated with 100-μm aluminum oxide particles at 1.0 bars pressure for 20 s at a distance of 10 mm. Afterwards all crowns and abutments were cleansed three times in an ultrasonic bath at 30 °C for 5 min each [15]. The first bath contained an antibacterial cleansing solution (FINEVO 01, Bredent GmbH & Co. KG, Senden, Germany), the second bath contained 80% ethylalcohol, and the third bath contained medically pure water (aqua dest.). In the second trial, a methacrylate cement (Panavia V5, Kuraray, Japan) was used for the cementation (Fig. 3). A second investigator (KB) not involved in the cementing process attempted to remove any cement residues. After setting of the zinc oxide non-eugenol cement or light curing of the resin cement, the excess was removed with a steel scaler (Hu Friedy Co., LLC, Tuttlingen, Germany) and super-floss (Procter & Gamble, Surrey, UK) until the investigator was convinced it had been completely cleaned. Once cleaned, constant vertical pressure on the crown was kept until the cement had fully set. Subsequently, the occlusal closing materials were removed, the abutment screw was unscrewed and the superstructure complex was dismounted for assessment (Fig. 4). A computerized planimetric method of cement assessment described by Linkevicius et al. was utilized [11]. All measurements were obtained by a single calibrated examiner (CF). Calibration was tested by double analysis of standardized digital photographs from 10 CAD/CAM abutments, with a one-week interval. The agreement coefficient was of 0.96, with a mean difference of 0.03 ± 0.92 (values ≥0.75 are considered excellent). The specimens were fixed on a custom-made device, to keep a standardized distance between the camera (Canon EOS D80, Tokyo, Japan) and the specimen. Digital photographs were taken from all four quadrants (mesial, buccal, distal and lingual) using a 100 mm macro objective lens. The images were imported and analyzed using Adobe Photoshop (Adobe Systems Ltd., Europe, Uxbridge, UK). For each image obtained from each quadrant, the circumference of the total crown-abutment surface was marked using a free line tool (lasso-tool) of the software. The number of pixels was recorded from the histogram option (Fig. 5). The same procedure was applied to the area covered with cement remnants following the contours of the excess cement. The ratio between the area covered with cement and the total surface area of the specimen was calculated. A surface of the specimen was considered as a statistical unit, therefore each specimen had four measurements, resulting in a sample size of 20 for each group. Statistical analyses were carried out using the program packages STATISTICA (STATSOFT, Tulsa, USA, version 9.1) and BiAS (Epsilon-Verlag, Frankfurt, version 11.02). Frequency distributions were used to characterize categorical variables. The Mann-Whitney-U-Test and the Kruskal-Wallis H-Test were used to compare independent groups for continuous variables. Significance was set at *p* < 0.05.

## Results

**Fig. 6:**
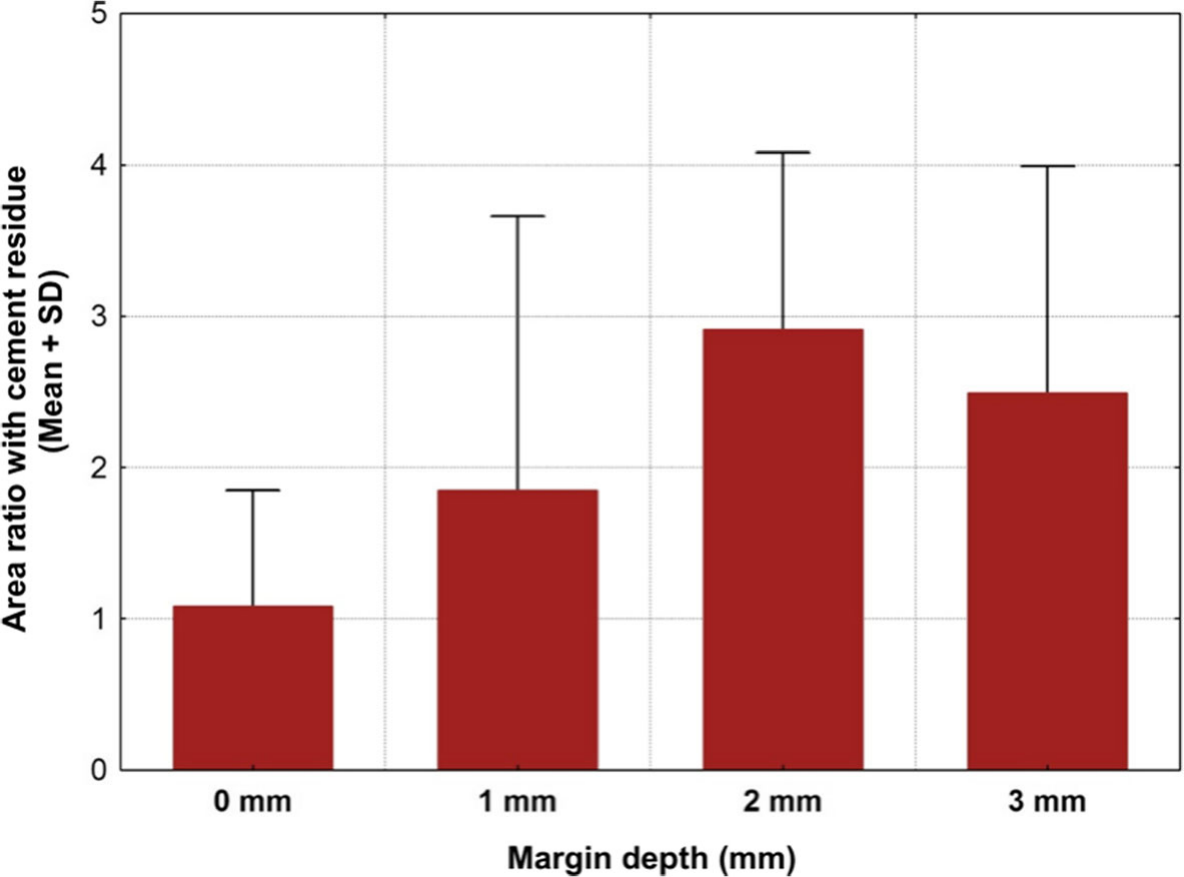
Total cement residues on lateral aspects: Temp Bond (Mean + SD).

**Fig. 7:**
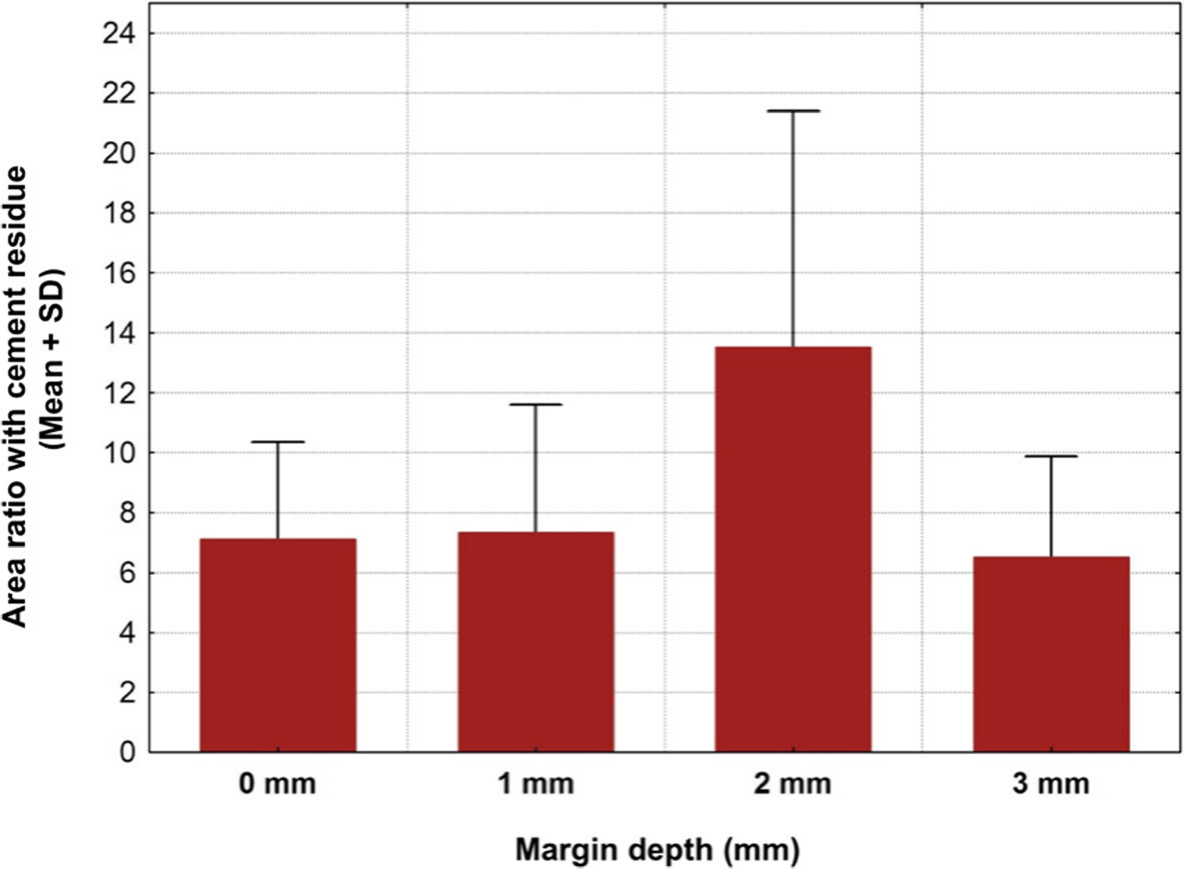
Basal cement residues: Temp Bond (Mean + SD)

**Fig. 8:**
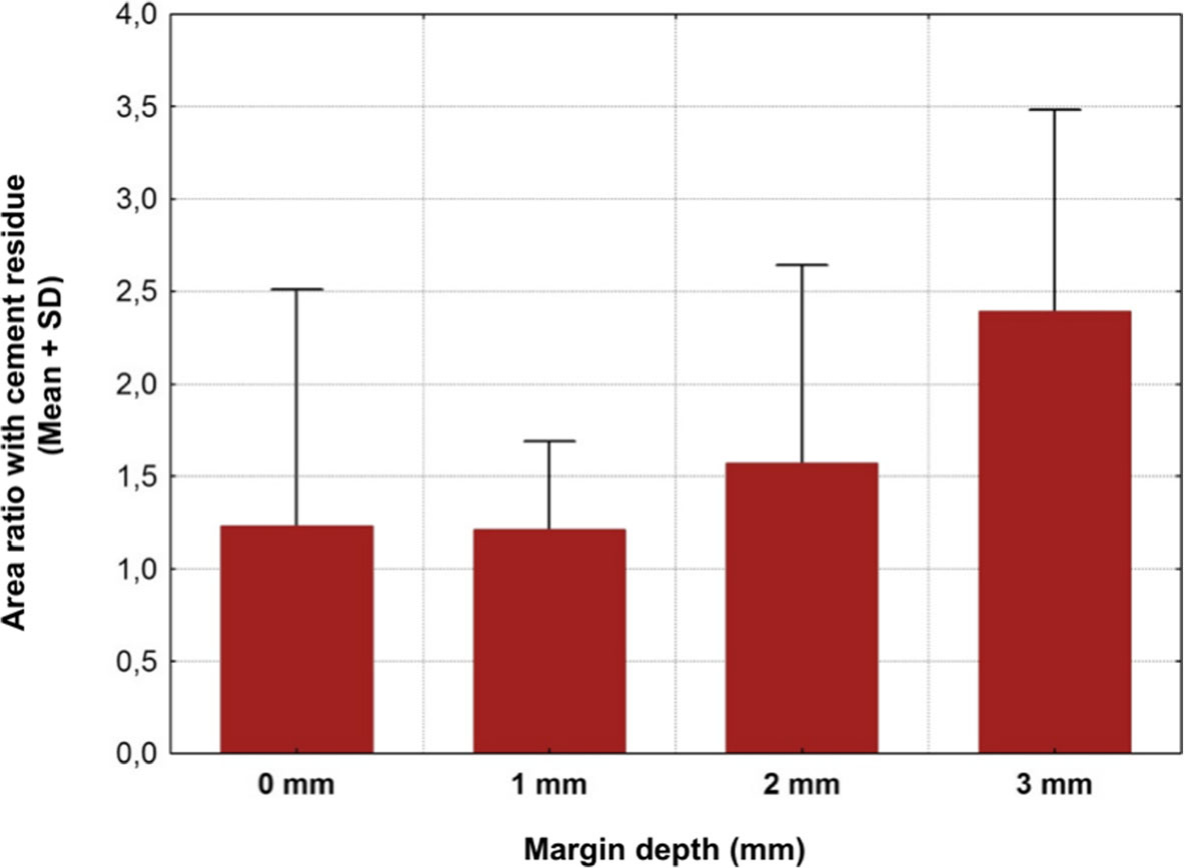
Total cement residues on lateral aspects: Panavia V5 (Mean + SD)

**Fig. 9:**
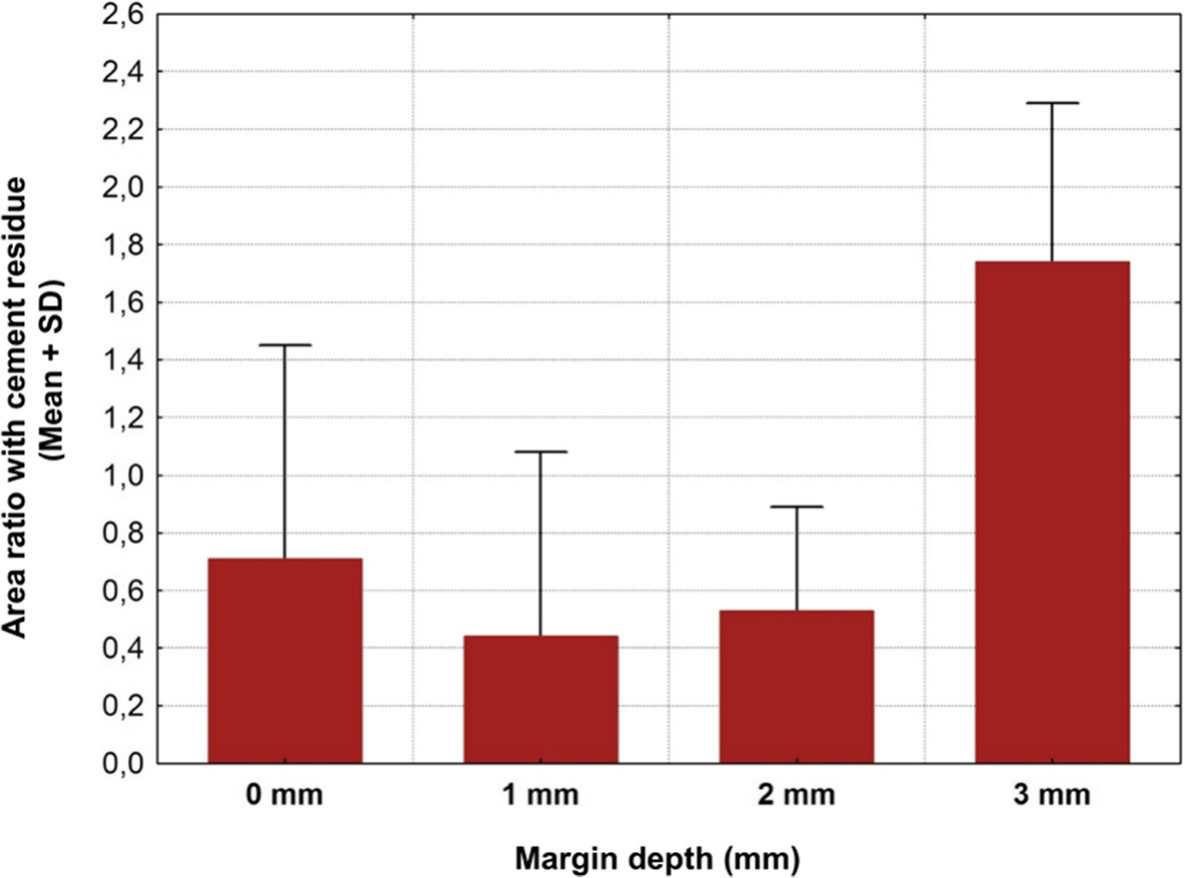
Lingual cement residues: Panavia V5 (Mean + SD

**Fig. 10:**
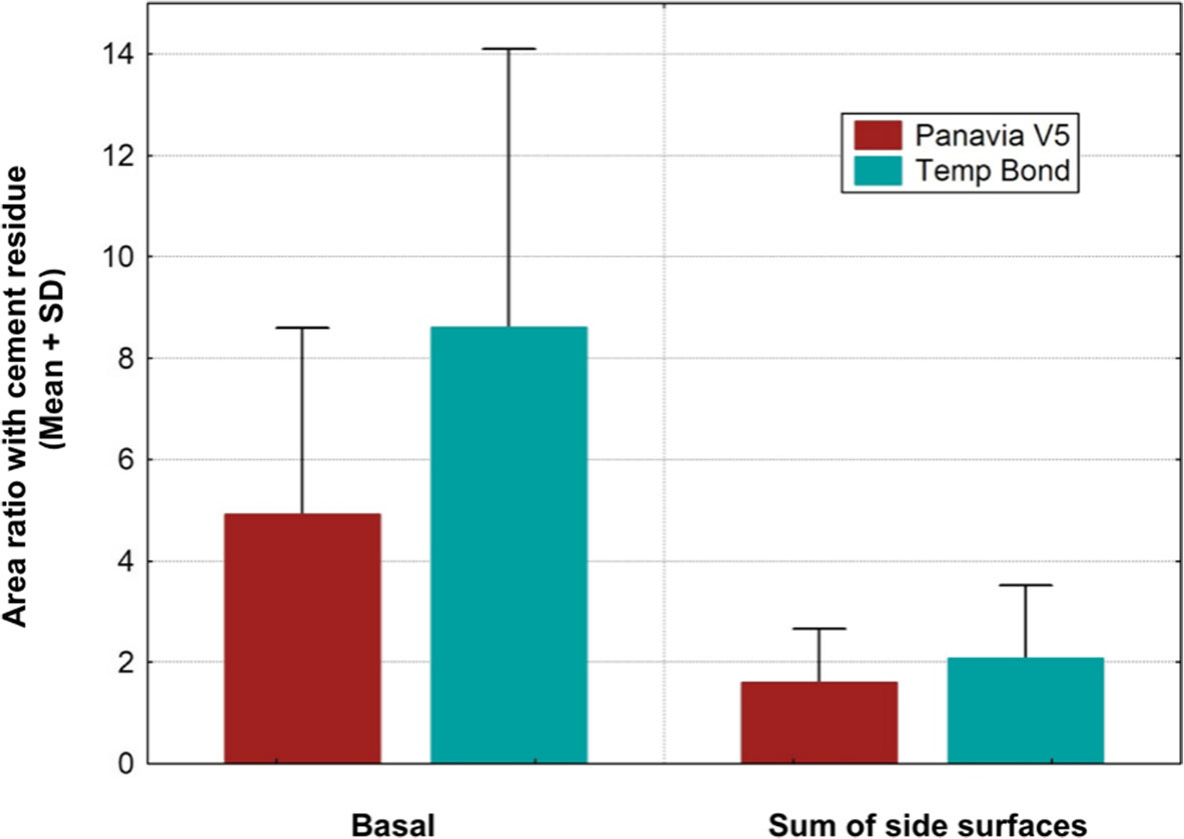
Summary of the comparison of the two cement types (%)

Cement remnants were found in almost every abutment area investigated. The amount of the remnants varied according to the depth of the crown-abutment margin. Excess cement was not only found at the margins of the crown-abutment complex, but also underneath (basal) the molar abutment itself, where no cleaning was possible due to its prominent emergence profile. The two types of cement were separately investigated and statistically analyzed. The summary of the total Temp Bond cement residues on the lateral aspects (mesial, buccal, distal and lingual) is shown in Table 1. Comparing the different margin levels, there was a clear tendency to increase the proportion of undetected Temp Bond residues on the lateral abutment surfaces with increasing margin depth from 0 to 2 mm (Fig. 6). Positioning the abutment shoulder from 2 to 3 mm depth, however, showed a slight decrease in excess cement. No statistical significance was found between the depth of margin and the frequency of remnants (total *p* = 0.1520) (Table 2). The Kruskal-Wallis-Tests for the individual surfaces revealed the following values: mesial *p* = 0.1106, distal *p* = 0.0581, buccal *p* = 0.061, lingual *p* = 0.1312. Although notable excess of Temp Bond could be identified at the basal surface of all investigated CAM/CAM molar abutments, their presence in relation to the individual abutment shoulder was not statistically significant (*p* = 0.336) (Tables 3 and 4) (Fig. 7). Comparing the four margin depths (0–3 mm subgingival) in the Panavia V 5 sample group, the buccal (*p* = 0.0860), mesial (*p* = 0.0922), distal (*p* = 0.9679), and basal surfaces (*p* = 0.9846) showed no statistical significance. The total cement residues of Panavia V 5 on the lateral abutment aspects are shown in Tables 5 and 6 and Fig. 8. However, the lingual surface demonstrated a statistical significance (*p* = 0.0291) at a margin depth of 3 mm subgingivally (Tables 7 and 8) (Fig. 9). Tables 9 and 10 show, separated by the cement type, the individual aspect, the sum of lateral aspects, and the amount of excess cement in percent irrespective of the gap depth. Table 11 displays the results for the comparison the two cement types utilized. The proportion of basal abutment aspects covered with cement residues was significantly smaller in the methacrylate cement samples (Panavia V5) with an average of 4.9 ± 3.7% compared to the temporary zinc oxide non-eugenol cement samples (Temp Bond) with an average of 8.6 ± 5.5% (*p* = 0.007) (Fig. 10). The difference in the mean sum of the lateral abutment surfaces affected by cement residues was not statistically significant (*p* = 0.398) (Table 11).

**Table 1:** Total cement residues on lateral aspects: Temp Bond (%)

Total Temp Bond cement residues on lateral aspects (%) (mesial, buccal, distal and lingual) (%)						
Margin depth	N	Mean	Median	Min	Max	SD
0	5	1.08	0.63	0.51	2.34	0.77
1	5	1.85	1.59	0.43	4.95	1.81
2	5	2.91	3.03	1.07	4.32	1.17
3	5	2.49	2.26	0.62	4.79	1.50

**Table 2:** Total cement residues on lateral aspects: Temp Bond (%)

Kruskal-Wallis-Test: H (3, *N* = 20) = 5.285714 p = 0.1520			
Margin depth	N	Rank total	Mean rank
0	5	32.00000	6.40000
1	5	46.00000	9.20000
2	5	73.00000	14.60000
3	5	59.00000	11.80000

**Table 3:** Basal cement residues: Temp Bond (%)

Basal Temp Bond cement residues (%)						
Margin depth	N	Mean	Median	Min	Max	SD
0	5	7.11	7.58	3.49	11.82	3.24
1	5	7.32	5.56	3.11	14.30	4.28
2	5	13.52	13.25	3.33	25.16	7.88
3	5	6.51	6.15	2.55	11.60	3.36

**Table 4:** Basal cement residues: Temp Bond (%)

Kruskal-Wallis-Test: H (3, N = 20) = 3.388571 *p* = 0.3355			
Margin depth	N	Rank total	Mean rank
0	5	48.00000	9.60000
1	5	48.00000	9.60000
2	5	73.00000	14.60000
3	5	41.00000	8.20000

**Table 5:** Total cement residues on lateral aspects: Panavia V5 (%)

Total Panavia V 5 cement residues on lateral aspects (mesial, buccal, distal and lingual) (%)						
Margin depth	N	Mean	Median	Min	Max	SD
0	5	1.23	0.68	0.42	3.47	1.28
1	5	1.21	1.33	0.42	1.66	0.48
2	5	1.57	1.13	0.91	3.46	1.07
3	5	2.39	2.31	1.26	3.78	1.09

**Table 6:** Total cement residues on lateral aspects: Panavia V5 (%)

Kruskal-Wallis-Test: H (3, N = 20) = 5.194286 *p* = 0.1581			
Margin depth	N	Rank total	Mean rank
0	5	34.00000	6.80000
1	5	51.00000	10.20000
2	5	49.00000	9.80000
3	5	76.00000	15.20000

**Table 7:** Lingual cement residues: Panavia V5 (%)

Panavia V 5 cement lingual residues (%)						
Margin depth	N	Mean	Median	Minimum	Maximum	SD
0	5	0.71	0.28	0.13	1.77	0.74
1	5	0.44	0.00	0.00	1.42	0.64
2	5	0.53	0.46	0.13	1.12	0.38
3	5	1.74	1.68	1.15	2.59	0.54

**Table 8:** Lingual cement residues: Panavia V5 (%)

Kruskal-Wallis-Test: H (3, N = 20) = 9.015686 *p* = 0.0291			
Margin depth	N	Rank total	Mean rank
0	5	50.00000	10.00000
1	5	32.00000	6.40000
2	5	43.00000	8.60000
3	5	85.00000	17.00000

**Table 9:** Comparison of the two types of cement: Panavia V5

Panavia V5	N	Mean	Median	Min	Max	SD
Basal: Cement residues (%)	20	4.91	3.53	0.05	13.19	3.69
Buccal: Cement residues (%)	20	0.94	0.73	0.00	3.23	0.83
Mesial: Cement residues (%)	20	2.05	1.65	0.00	6.97	1.96
Lingual: Cement residues (%)	20	0.85	0.70	0.00	2.59	0.76
Distal: Cement residues (%)	20	2.58	2.08	0.00	6.97	1.66
Sum of lateral aspects: Cement residues (%)	20	1.60	1.30	0.42	3.78	1.06

**Table 10:** Comparison of the two types of cement: Temp Bond

Temp Bond	N	Mean	Median	Min	Max	SD
Basal: Cement residues (%)	20	8.61	7.49	2.55	25.16	5.48
Buccal: Cement residues (%)	20	1.41	1.09	0.04	4.20	1.27
Mesial: Cement residues (%)	20	2.40	2.11	0.00	6.23	1.89
Lingual: Cement residues (%)	20	1.03	0.89	0.00	4.22	0.96
Distal: Cement residues (%)	20	3.60	3.24	0.58	8.95	2.49
Sum of lateral aspects: Cement residues (%)	20	2.08	1.88	0.43	4.95	1.44

**Table 11:** Summary of the comparison of the two types of cement (%)

Mann-Whitney U-Test						
	Rank total	Rank total	U	N	N	2*incl.
	Temp Bond	Panavia V5		Temp Bond	Panavia V5	exact p
Basal: Cement residues (%)	508.0000	312.0000	102.0000	20	20	0.007331
Buccal: Cement residues (%)	449.0000	371.0000	161.0000	20	20	0.301253
Mesial: Cement residues (%)	439.0000	381.0000	171.0000	20	20	0.444964
Lingual: Cement residues (%)	430.5000	389.5000	179.5000	20	20	0.583114
Distal: Cement residues (%)	453.0000	367.0000	157.0000	20	20	0.253380
Sum of lateral aspects: Cement residues (%)	442.0000	378.0000	168.0000	20	20	0.398302

## Discussion

Biological properties of the interface between implant abutment and surrounding tissues are of critical importance for long-term success [17]. Cement remnants of fixed implant-supported restorations have been associated with clinical and radiographic signs of peri-implantitis [6, 13, 18]. Numerous in vitro and in vivo studies demonstrated that the depth of the crown-abutment interface of stock abutments negatively influence the practitioner’s ability to remove cement remnants [8, 10–12]. It has been claimed that the application of computer-aided design and computer-aided manufacturing (CAD/CAM) facilitates the formation of an anatomical abutment design with a natural emergence profile and a proper spatial outline at the cervical margin [19]. The flexibility in designing the submucosal part of the custom abutment and the positioning of the shoulder finish line has been suggested to reduce the challenges of undetected excess cement [20]. Unlike most previous studies [6, 10–12], the present investigation analyzed the quantity and depth of remnants around custom CAD/CAM abutments with a convex emergence profile at different regions of a molar crown-abutment complex. The results revealed cement remnants in every depth of the crown-abutment complex and in almost every investigated area. The amount of cement left was influenced by the location of the crown-abutment margin. Although no statistical significance was found between the depth of margin and the presence of remnants, an increase in remnants was detected when the crown-abutment margin was located more submucosally. Deep crown-abutment margin positions (deeper than 1 mm below mucosa) increased the risk of residual cement. Excess cement was more difficult to remove utilizing a scaler and super-floss at oral and approximal surfaces than other surface areas. It could be demonstrated, that cement remnants were not only found at the margins of the crown-abutment complex, but also at the basal surface of the abutment. Therefore, it is essential to exercise the utmost care when cementing crowns to CAD/CAM molar abutments with a prominent convex emergence profile. These findings are in concordance with recent research and clinically relevant as they uncover the critical areas where cement remains even after careful removal attempts [8, 11, 12, 14, 21, 22].

In the clinical context, a mismatch between the digitally planned margin of a CAD/CAM-customized abutment, and its intraoral position after delivery and functioning can be found [23, 24]. The use of standard impression copings or implant scan bodies with a circular diameter produces inconsistency within the emergence profile. It may begin with a collapse of the supra-implant mucosa during intra-oral optical or conventional impression taking and then result in computer-generated mismatch of the position and contour of the abutment shoulder. Hence, there is a risk of discrepancy between the position of the free mucosal margin in a digital image and its actual position. In a recent clinical study Pietruski et al. [25] examined the concordance between the virtual planned and the clinical position of an abutment shoulder against the mucosal margin and their actual position. Although for the majority of all cases (a total of 257 abutments) soft tissue stability or growth could be confirmed favouring the use of CAD/CAM abutments, in 20% of cases, soft tissue height reduction was demonstrated with unfavorable abutment shoulder display. In order to avoid the risk of soft tissue deficiency, the authors recommended to set the abutment shoulder slightly deeper submucosally than the CAD software routinely recommends.

There are several limitations associated with this in vitro study. Therefore, the results obtained can not be directly translated into the clinical context. Beside its in vitro nature, the small sample size, although balanced using proper statistical analysis, might limit the generalization of the study outcomes. The statistically significant result for an increased presence of methacrylate cement (*p* = 0.0291) at the lingual abutment surface must be regarded with caution. Even though the cementation and subsequent cleaning procedures were kept as close as possible to clinical reality, the in vitro nature of the study setup might limit a clinical generalization of the outcomes. A master cast with a silicone gingiva mask cannot entirely replicate the nature of the peri-implant sulcus and its interaction with the submucosal anatomy of a convex abutment configuration. In addition, clinical data suggest that temporary cements such as zinc oxide non-eugenol materials are more likely to be washed out by sulcus fluids than resin based cements [26, 27]. Further clinical research is needed to confirm or disprove these results. One of the major difficulties in cementing the crowns was the standardization of the amount of cement. Alternatively, a micro brush should have be considered to apply a uniform layer of cement or automatic pipettes to avoid initial disparities between the groups. Another drawback relates to the surface measurement of cement residues. Since cement volume and weight were not assessed, data analysis was more complex. In some cases, after the attempt of cement removal, small amounts of cement could be spread over a larger area, compromising the reliability of the results. In order to be able to compare the results of the present study with those of earlier in vitro and clinical trials [11, 12], it was decided to use digital photography for evaluation. Instead of using digital photographs to analyze cement excess, scanning electron microscopy (SEM), with significantly higher image resolution, would have enabled a more objective assessment of cement residue. A SEM assessment might have detected higher cement volumes and therefore altered the results. Although the current results cannot be directly extrapolated to the clinical situation, the potential occurrence of adverse effects caused by excess cement should be considered when making clinical decisions.

The impact of cement remnants in the development of peri-implantitis is still discussed controversially. Excess cement in subgingival areas is described as an “artificial calculus” and may have a similar irritating effect as calculus on periodontally involved teeth [28]. A multicenter 3-year prospective study reported that peri-implant soft tissues reacted more favorably to screw-retained crowns when compared with cement-retained restorations [29]. A recent clinical trial compared cemented versus screw-retained single implant-supported ceramic crowns in terms of histological, microbiological and radiological outcome measures 6 months after insertion [30]. Although both types of reconstructions resulted in a similar radiological and clinical outcome, the results displayed that cemented restorations were associated with more inflammatory cells and more patients were diagnosed with periodontal pathogens. In contrast Blanes et al. [31] showed that peri-implant tissues around cemented restorations were not more inflamed when compared to tissues around screw-retained prostheses.

It should be emphasized, however, that the excess of cement is just one of several potential factors causing tissue inflammation and the development of peri-implantitis. While peri-implantitis represents a predominantly plaque-induced inflammatory condition [32], certain local factors may be associated with this biologic complication, as they involve plaque retention. Recent longitudinal and cross-sectional trials have investigated additional parameters that could promote the onset of adverse conditions and encourage the transformation of physiologic bone loss to peri-implant disease [33–35]. As the patient’s compliance in supportive peri-implant maintenance plays an important factor in determining the chance of developing peri-implant disease [36], smoking and alcohol consumption are supposed to be potential contributing factors [32, 37]. Foreign body reactions to alloplastic grafting materials [35], varying soft and hard tissue composition [38], or improper three-dimensional implant positioning [39], might also predispose to the presence of disease. Regardless of the implant positioning and placement protocol, vertical and horizontal bone remodeling has been described [40, 41] which in some cases may result in minimal thread exposure followed by the adherence of pathogenic bacteria, which in turn promote bone resorption. Implant surface morphology [42], contamination of the inner part of the implant connection [43] and contamination due to the laboratory workflow [15, 16] might represent other pathogenic pathways for peri-implant disease.

Canullo et al. [44] demonstrated in a recent cross-sectional clincal study that although symptoms of peri-implantitis are always a plaque-induced inflammatory entity, certain prosthetic (eg, inadequate superstructure design, incorrect distribution of prosthetic loading), surgical (eg, implant malpositioning, failed bone reconstruction), or biomechanical (eg, overloading) factors might be associated with this clinical phenomenon. Interestingly, in the aforementioned clinical trial involving 554 patients and 1507 dental implants, the second most common determinant of peri-implantitis was implant width. The authors assumed that bone grafting and/ or higher compression force created during the drilling sequence for wider implant placement might inject another contributing factor for developing peri-implantitis.

Until now, a fixed implant supported restoration could either be cemented or screw-retained. Recently, a cone-in-cone morse taper connection between the abutment and the crown has been alternatively introduced to retain implant-retained definitive fixed dentures (FDPs). The frictional connection eliminates the use of cements or screws, allowing for easy retrieval of the restorations with regular maintenance. This restorative approach, named the “Acuris™ Conometric Concept” (Dentsply Sirona Implants, Bensheim, Germany), has been used to retain hybrid acrylic-composite [45], monolithic lithium disilicate [46, 47] and monolithic zirconia [48] implant restorations in the posterior region. The authors reported favorable mid-term results with high implant survival, stable hard and soft tissues, and few prosthetic complications.

## Conclusions

Given the results obtained in the present in vitro investigation, the margin of CAD/CAM molar abutments should be located as coronally as possible to minimize the amount of cement remnants. If this ideal margin location is not feasible due to esthetic concerns, it cannot be recommended to place the margin of molar abutments deeper than 1.5 mm in the approximal and oral regions.

## Data Availability

The datasets used and/or analysed during the current study are available from the corresponding author on reasonable request.
